# How parents express their worry in calls to a medical helpline: a mixed methods study

**DOI:** 10.1186/s12875-022-01680-4

**Published:** 2022-04-15

**Authors:** Caroline Gren, Maria Kjøller Pedersen, Asbjørn Børch Hasselager, Fredrik Folke, Annette Kjær Ersbøll, Dina Cortes, Ingrid Egerod, Hejdi Gamst-Jensen

**Affiliations:** 1 Department of Pediatrics and Adolescent Medicine, Copenhagen University Hospital – Amager and Hvidovre, Copenhagen, Denmark; 2 Department of Clinical Medicine, University of Copenhagen, Copenhagen, Denmark; 3 Department of Nursing and Nutrition, University College Copenhagen, Copenhagen, Denmark; 4 Department of Pediatrics and Adolescent Medicine, Copenhagen University Hospital – Herlev and Gentofte, Copenhagen, Denmark; 5 Copenhagen University Hospital – Copenhagen Emergency Medical Services, Copenhagen, Denmark; 6 Department of Cardiology, Copenhagen University Hospital – Herlev and Gentofte, Copenhagen, Denmark; 7 National Institute of Public Health, University of Southern Denmark, Copenhagen, Denmark; 8 Department of Intensive Care, Copenhagen University Hospital – Rigshospitalet, Copenhagen, Denmark; 9 Department of Clinical Research, Copenhagen University Hospital – Amager and Hvidovre, Copenhagen, Denmark; 10 Emergency Department, Copenhagen University Hospital – Amager and Hvidovre, Copenhagen, Denmark

**Keywords:** Telemedicine, Telephone triage, Out-of-hours care, After hours, Telenursing, Primary care, Pediatrics, Mixed methods study, Worry, Parents, Denmark

## Abstract

**Background:**

Telephone triage is used globally in out-of-hours primary care, to prioritize who needs urgent assessment. Even though children rarely are severely ill, calls about sick children are among the most prevalent, mainly due to parental worry. Pediatric calls are considered challenging, as the call-handler must rely on parents’ second-hand information. We aimed to investigate if parents’ worry can be used as a predictor of severe illness, and if the content of the calls varies between different grades of worry.

**Methods:**

In a convergent mixed methods study design we asked patients to rate their degree-of-worry before talking to a call-handler. We used quantitative data of degree-of-worry, triage- and patient outcome in pediatric calls (*n* = 2857), and the qualitative content from 54 calls with subsequent hospitalization ≥24 h.

**Results:**

High degree-of-worry was associated with hospitalization ≥24 h (OR 3.33, 95% CI 1.53–7.21). Qualitative findings both confirmed and expanded knowledge of degree-of-worry. Worry was the predominant cause for contact overall, and was mainly triggered by loss-of-control. In calls with high degree-of-worry, the prevalence of loss-of-control was especially high, and the parents had additionally often contacted healthcare services recently. Parents with a foreign accent often rated their worry as high, and these callers were often ignored or interrupted. Calls with low degree-of-worry seemed to occur early during the disease.

**Conclusion:**

High degree of parental worry was associated with severe illness. At the end of calls, call-handlers should ensure that the parent has regained control of the situation to reach increased reassurance and to prevent renewed unnecessary contact. Safety-netting is crucial, as many parents made contact early during the illness and deterioration may develop later. The scoring of parental degree-of-worry may be used as an indicator of potentially severe illness and can easily be implemented at out-of-hours call-centers globally.

**Trial registration:**

Original study registered at clinicaltrials.gov (NCT02979457).

## Introduction

Telephone triage is used globally to counteract crowding in emergency departments during out-of-hours (OOH) periods, and as a method to lessen the workload of primary care physicians [1]. The set-up of call centers varies greatly, but the telephones are often staffed by nurses, with varying backgrounds. Telephone triage is considered difficult, and even more so when calls concern children, as the call-handler must rely on second-hand information conveyed by the caregiver, usually a parent, without visual input [2, 3]. The parent must interpret signs that are often unspecific and from a non-verbal young child, and communicate this to the call-handler, after deciding what might be relevant to pass on. Pediatric calls are highly prevalent at OOH medical helplines [4, 5] even though acutely ill children rarely are severely ill: only approximately 1% of children assessed acutely in general practices (GP) or pediatric emergency departments (PED) have a serious illness [6, 7]. The majority of pediatric contacts in OOH care are related to infections, such as fever, symptoms from the upper respiratory tract, and ear infection [4, 8, 9]. The large number of OOH calls concerning children is most often caused by parental worry [10–12], but other motives for contact range from convenience and logistical reasons, to fear of doing the wrong thing and need for reassurance and sharing of responsibility [13–16]. Fever, being first-time parents, and lack of social support are factors also associated to experiencing a need to contact health services [14, 15, 17, 18]. Thus, parents’ expectations when contacting OOH care are not necessarily medication or cure, but often rather reassurance and guidance [15–19].

It is unknown what value the parents’ sense of worry and urgency could have in relation to predicting severe illness among the large number of mildly ill children. A prospective study with almost 4000 acutely ill children assessed in Dutch general practices registered symptoms and outcome and constructed classification trees to create a triage tool to identify the most severely ill children. The parents’ feeling of “illness is different” was highly usable, although less important than the clinician’s gut feeling of “something is wrong” [20]. Another study investigated parents’ self-referral of febrile children to a PED, of whom 25% required treatment, tests and/or admission as opposed to 43% GP-referred children [21]. Studies performed at the OOH Medical Helpline 1813 (MH1813) in Copenhagen, Denmark, showed that callers from the general population were able to score their degree-of-worry (DOW) before talking to a call-handler and that there was a strong dose-response relationship between DOW and acute hospitalization ≥24 h. However, there was no difference in triage outcome between calls where the call-handlers had access to the callers’ DOW score and in calls without DOW score [22–24]. It is unknown if the association between DOW and hospitalization also applies for children. In this study, we aimed to explore parental worry about acutely ill children in calls to an OOH medical helpline, by investigating 1) the association between DOW and triage response and acute hospitalization with a duration ≥24 h, where the duration of hospitalization is used as a proxy for severe illness, and 2) how the qualitative content in parents’ communication with call-handlers can be used to expand the quantitative results and make them more useful in clinical situations. We used a mixed methods design to explore our understanding of the findings and to enlighten the complex phenomenon of parental worry from qualitative and quantitative perspectives.

## Methods

### Design

**Fig. 1:**
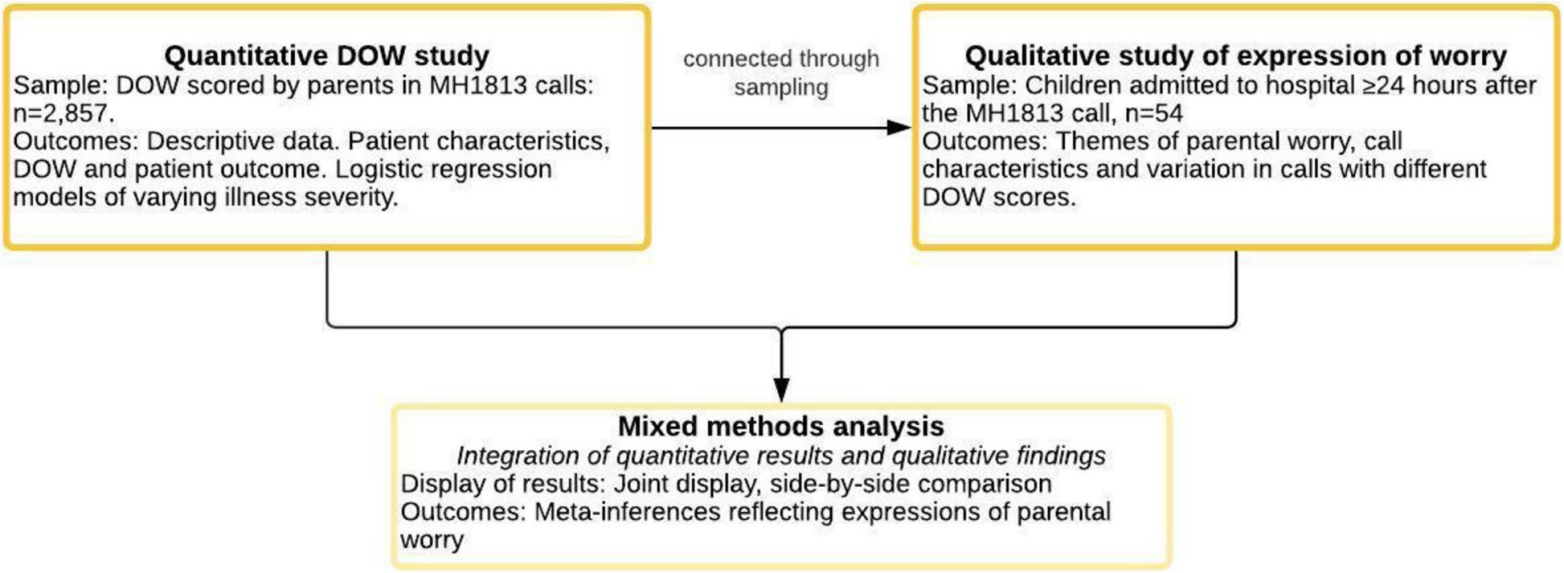
Convergent mixed methods study design

We used a convergent mixed methods study design (i.e. qualitative and quantitative data are collected and analyzed during a similar timeframe) with equal weighting of strands (i.e. equal weight of qualitative and quantitative data), Fig. 1 [25]. The strands were connected through sampling, where the quantitative data informed the sampling of the qualitative strand. The quantitative and qualitative results were integrated (i.e. combined) in the analysis and display of results [26].

### Setting

Between January 24th and February 9th, 2017, callers to the MH1813 in the Capital Region of Denmark were invited to participate in a study about worry in acute illness. Eleven thousand three hundred forty callers consented to participate (hereafter referred to as the DOW population). They were asked to rate their DOW concerning the problem they called about on a scale from 1 to 5, where 1 was minimally worried and 5 was extremely worried, while waiting in line. Gamst-Jensen et al. have published further details regarding the DOW-study [22–24]. The present study focuses exclusively on the data collected from the pediatric population in the DOW-study.

MH1813 is part of the Emergency Medical Services (EMS) Copenhagen. MH1813 is an OOH helpline for non-emergent acute injury and illness that cannot wait until GP offices open. EMS Copenhagen is also accessible via another telephone number (1-1-2), directed at life-threatening illness and injury. EMS Copenhagen has a catchment population of approximately 1.8 million citizens, and MH1813 receives about 1 million calls annually. Approximately 25% of the calls concern children, of whom 40% are referred to assessment at a hospital. MH1813 is staffed by registered nurses and physicians, the majority (80%) being nurses. Both nurses and physicians come from varying backgrounds, and as such, are not required to have pediatric specialization for handling pediatric calls, A few call-handlers, however, have pediatric background. Physicians function as either second-level support or as primary call-handlers. MH1813 is usually the main place of employment for the nurses, but a secondary job for physicians. Before taking calls independently, the nurses receive a 6-week theoretical and practical introduction. The physicians receive a shorter introduction, mainly focused on the software systems used. Furthermore, nurses only are obligated to use a symptom-based decision-support tool, guiding them through relevant questions, diagnoses and triage outcomes. The tool is developed in-house and not externally validated.

The call-handlers basically have two triage responses at the end of the call: the patient can stay at home with self-care guidance (and, if necessary, the patient can contact the GP the next workday or MH1813 again) or the patient is referred to hospital for assessment at an urgent care clinic or emergency department. If needed, calls are forwarded to the EMS ambulance services. The public is strongly encouraged to call either the MH1813 or 1–1-2 before accessing a hospital OOH. As for most health services in Denmark, the MH1813 service is paid through taxes and is free-of-charge.

### Study population

The present study population is a subsample of the DOW population including all children aged ≤11 years. This inclusion criterium was chosen as it was the cut-off for assessment at the regional pediatric urgent care clinics. Exclusion criteria were calls concerning injuries, and calls registered as “other reason for calling”, as these most often do not directly concern illness, e.g. prescription renewal, or calls from other regions.

Patients were classified according to the outcome within 48 h after the call, as a proxy for illness severity: group 1, patients receiving telephone consultation (TC); group 2, patients assessed at a hospital for face-to-face consultation (FTF), but not hospitalized ≥24 h; and group 3, patients hospitalized ≥24 h. This classification allowed us to study the association of DOW to different degrees of illness severity.

Only the call made in connection with DOW-rating was included, which means that the caller might have called several times regarding the same episode of illness, but only one call was included in the DOW study. However, if a later call within 48 h of the initial call resulted in hospital admission, the patient was classified with this outcome. All available patients were included in the quantitative analyses (*n* = 2857). Purposive sampling was used for the qualitative study, and only patients from group 3 were sampled (*n* = 55) as these represent the most severely ill children where we would expect to find the most manifest content of DOW.

### Data collection

In addition to DOW, data were derived from two sources linked by personal identification number. 1) The internal data registration system at MH1813 has an incorporated triage tool used by the call-handlers, from which we collected data on personal identification number, age, gender, reason for calling as registered by the call-handler; triage response (TC or FTF) and voice logs, i.e. the recorded calls, used in qualitative analysis. 2) Information on date and time for hospital admission along with discharge time and primary diagnosis was retrieved from the Danish National Patient Register [27].

### Data analysis

#### Quantitative analysis

Descriptive analysis of age, gender, reason for calling, triage response, DOW and hospital diagnosis was conducted using frequency distributions (numbers and percentages), median and interquartile range (IQR). Logistic regression analysis was used to examine association between DOW and probability of hospitalization, and between DOW and triage response. We conducted both an unadjusted analysis and an analysis adjusted for child’s gender and age. Ordinal logistic regression analysis was conducted for the association between DOW and patient outcome (groups 1–3), also adjusted for gender and age. Age was included as a categorical variable (ages 0–2, 3–5 and 6–11). Due to few children hospitalized ≥24 h, we changed DOW from five to three categories in the regression analyses (1 = DOW 1 + 2; 2 = DOW 3, 3 = DOW 4 + 5). Results are presented as odds ratios (OR) with 95% confidence intervals (CI). The statistical analyses were made in SAS Enterprise Guide 7.1 (SAS Institute, Cary, NC, USA). The reporting of the quantitative study is in accordance with STROBE guidelines [28].

#### Qualitative analysis

Calls in group 3 were transcribed verbatim and analyzed inductively using content analysis (*n* = 54). Content analysis was chosen as we had little contextual knowledge about the parents, which limited the level of interpretation. Content analysis permits the transformation of qualitative data into quantitative measures, such as the relative distribution of codes in different subgroups. Each call was considered a unit of analysis. The process of analysis followed the steps described by Graneheim and Lundman [29], where meaning units are coded and the codes subsequently are arranged in categories reflecting common manifest content. Lastly, themes are created, which are considered as “a thread of an underlying meaning through condensed meaning units, codes or categories, on an interpretative level. A theme can be seen as an expression of the latent content of the text.” [29]. We strived to make the categories internally homogenous and externally heterogenous, whereas themes did not need to be mutually exclusive. The inductively derived codes were reassessed by researcher triangulation. Transcription, coding and initial creation of categories and themes were performed by primary investigator CG (MD) and supervised by MKP, HGJ and IE (RNs, Ph.D.s). After discussion of findings and revision of codes and themes, we identified four themes, with seven categories and 21 codes. Analysis was carried out in NVivo 12 Plus (QSR International Pty Ltd., 2018). We used the COREQ 32-item checklist to assess the reporting of the qualitative study [30].

#### Mixed methods analysis

The qualitative and quantitative data were analyzed separately, followed by an iterative mixed methods analysis [25]. We initially transformed qualitative data into quantitative data, i.e. number of times codes were used, and how they were distributed within the three patient outcome groups according to DOW score (low, medium, high), to explore differences between DOW-groups. Integration between qualitative and quantitative data occurred at several levels [26]. At the method level, it was achieved through connection, as qualitative informers originated from the quantitative study population. Integration at the analysis and reporting level were enabled through data transformation and joint display (i.e. visual display integrating qualitative and quantitative findings). Thus, the findings of the data strands were merged in a side-by-side joint display to provide insights into the complex aspect of parental worry [31]. Meta-inferences were used to assess the fit between quantitative and qualitative findings and could result in confirmation (findings reinforced each other), expansion (findings expanded insights) or discordance (findings contradicted each other). The reporting of the study is in accordance with GRAMMS [32].

### Validity and reliability

Several types of validity, or types of legitimation, important to ensure the quality of mixed methods research have been suggested [33]. Especially interesting for this study is weakness minimization legitimation, where the large number of quantitative observations yield statistically stable results, but possibly rather unnuanced, which was complemented with the use of actual conversations providing subjective distinctions. Also, as we used actual calls, and not pre-planned interviews, we probably reached a higher degree of differences in stakeholders and thus representations of different viewpoints, i.e. sociopolitical legitimation. Commensurability approximation legitimation was gained through the research group consisting of persons of both sexes with different professions, medical backgrounds and varying experiences with quantitative and qualitative research.

## Results

### Quantitative strand

**Fig. 2:**
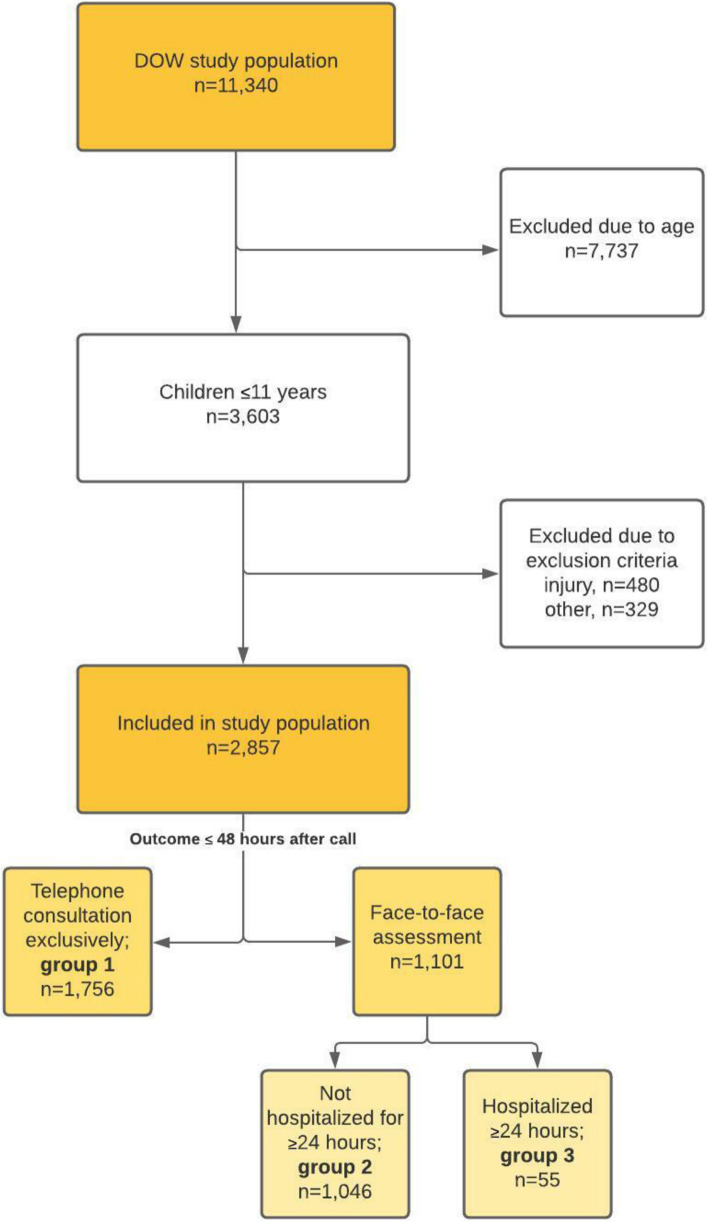
Flowchart of study participants

We included 2857 patients from the DOW study population, Fig. 2.

Of the 2857 included children in the study 1245 (43.6%) were referred to FTF consultation, but at 48 h after the call only 1101 children had been at a hospital, i.e. some parents had chosen not to take their children to the hospital after all.

Out of the total study population, 1046 (36.6%) were assessed at a hospital or hospitalized < 24 h and 55 (1.9%) ≥24 h, i.e. totally 1101 patients. Median age was similar in all three groups, but the youngest and oldest children were relatively more common in group 3 (Table 1).

**Table 1:** Patient characteristics, overall and stratified by patient outcome. Values are expressed as numbers (n) and percentages (%), unless stated otherwise

	Study population (***n*** = 2857)	Group 1: Telephone consultation; 61.5% (***n*** = 1756)	Group 2: Assessed at hospital, but not hospitalized ≥ 24 h; 36.6% (***n*** = 1046)	Group 3: Hospitalized ≥ 24 h; 1.9% (***n*** = 55)
**Gender**				
Boys	1.523 (53.5%)	933 (53.1%)	562 (53.7%)	28 (50.9%)
**Age**				
Median (IQR)	2.0 (1–5)	2.0 (I1–5)	2.0 (1–6)	1.0 (0–7)
0–2 years	1501 (52.5%)	901 (51.3%)	566 (54.1%)	34 (61.8%)
3–5 years	658 (23.0%)	437 (24.9%	216 (20.7%)	5 (9.1%)
6–11 years	698 (24.4%)	418 (23.8%)	264 (25.2%)	16 (29.1%)
**DOW**				
Median (IQR)	3.0 (2–4)	3.0 (2–3)	3.0 (2–4)	3.0 (3–4)
1	297 (10.4%)	218 (12.4%)	77 (7.4%)	2 (3.6%)
2	634 (22.2%)	407 (23.2%)	220 (21.0%)	7 (12.7%)
3	1175 (41.1%)	721 (41.1%)	432 (41.3%)	22 (40.0%)
4	503 (17.6%)	266 (15.2%)	220 (21.0%)	17 (30.9%)
5	248 (8.7%)	144 (8.2%)	97 (9.3%)	7 (12.7%)
**Aggregated DOW**				
Low (DOW 1 + 2)	931 (33%)	625 (36%)	297 (28%)	9 (16%)
Medium (DOW 3)	1175 (41%)	721 (41%)	432 (41%)	22 (40%)
High (DOW 4 + 5)	751 (26%)	410 (23%)	317 (30%)	24 (44%)
**MH1813 triage response**				
Telephone consultation	1612 (56.4%)	1488 (84.7%)	111 (10.6%)	13 (23.6%)
Face-to-face consultation	1245 (43.6%)	268 (15.3%)	935 (89.4%)	42 (76.4%)
**Reason for calling**				
URTI including ear symptoms	460 (22.1%)	285 (22.0%)	173 (23.3%)	2 (4.6%)
Fever/influenza	399 (19.2%)	257 (19.9%)	134 (18.0%)	8 (18.6%)
Gastrointestinal symptoms	268 (12.6%)	169 (13.1%)	88 (11.8%)	11 (25.6%)
Cardiopulmonary symptoms	279 (13.4%)	114 (8.8%)	153 (20.6%)	12 (27.9%)
Counselling on medication or vaccination	84 (4.0%)	77 (6.0%)	7 (0.9%)	–
Skin symptoms	162 (7.8%)	119 (9.2%)	42 (5.6%)	1 (2.3%)
Unspecified symptoms	140 (6.7%)	102 (7.9%)	35 (4.7%)	3 (7.0%)
Eye symptoms	156 (7.5%)	104 (8.0%)	51 (6.9%)	1 (2.3%)
Urogenital symptoms	41 (2.0%)	13 (1.0%)	27 (3.6%)	1 (2.3%)
Mouth/teeth/nose symptoms	33 (1.6%)	23 (1.8%)	9 (1.2%)	1 (2.3%)
Musculoskeletal symptoms	20 (1.0%)	11 (0.9%)	9 (1.2%)	–
Guidance/worry	20 (1.0%)	13 (1.0%)	5 (0,7%)	2 (4.6%)
Central nervous system symptoms	18 (0.9%)	6 (0.5%)	11 (1.5%)	1 (2.3%)
Missing	777 (27.2%)	463 (26.4%)	302 (28.9%)	12 (21.8%)
**Primary hospital diagnoses, ICD10-chapters**				
Respiratory tract diseases	374 (34.0%)	n/a	347 (33.2%)	27 (49,1%)
Symptoms and abnormal findings not classified elsewhere + Factors influencing health status and contact with health services	189 (17.2%)		179 (17.1%)	10 (18.2%)
Infection, unspecified	180 (16.4%)		176 (16.8%)	4 (7.3%)
Eyes and ears diseases	158 (14.4%)		156 (14.9%)	2 (3.6%)
Injury or external cause of illness	91 (8.3%)		89 (8.5%)	2 (3.6%)
Skin diseases	37 (3.4%)		36 (3.4%)	1 (1.8%)
Gastrointestinal diseases	34 (3.1%)		28 (2.7%)	6 (10.9%)
Urogenital diseases	19 (1.7%)		18 (1.7%)	1 (1.8%)
Endocrine diseases	5 (0.5%)		4 (0.4%)	1 (1.8%)
Musculoskeletal diseases	4 (0.4%)		4 (0.4%)	–
Hematopoietic diseases	3 (0.3%)		3 (0.3%)	–
Perinatal illness	3 (0.3%)		3 (0.3%)	–
Malformation and anomaly	2 (0.2%)		2 (0.2%)	–
Nervous system diseases	2 (0.2%)		1 (0.1%)	1 (1.8%)

*IQR* interquartile range, *DOW* degree-of-worry, *MH1813* Medical Helpline 1813, *TC* telephone consultation, *URTI* upper respiratory tract infection, *ICD10* International Classification of Diseases, 10th edition, *n/a* not applicable

Median DOW was 3.0 in the three groups but with varying IQR due to lower and higher DOW grades being more common in group 3. Even so, more than half of the parents in group 3 scored their DOW to low or medium and only 76.4% of these children were initially referred to consultation at hospital. Furthermore,

symptoms and diagnoses varied considerably between the groups. The reason for calling as registered by the call-handlers showed a high prevalence of upper respiratory tract infections such as cold or ear infections (22.0%), and fever (19.9%) in the TC group. In group 3, symptoms from the cardiopulmonary system (27.9%; mainly trouble breathing) and the gastrointestinal tract (25.6%) dominated. In both groups 2 and 3, i.e. among patients who had been at a hospital and thus had received a diagnosis, respiratory diagnoses were most common, followed by unspecific symptom codes (ICD-10 R and Z codes). Thereafter, the diagnoses differed, reflecting less severe symptoms in group 2, with diagnoses representing infection (mainly unspecified viral infections) and infections of eyes and ears dominating. Gastrointestinal diagnoses were more prevalent in group 3.

**Fig. 3:**
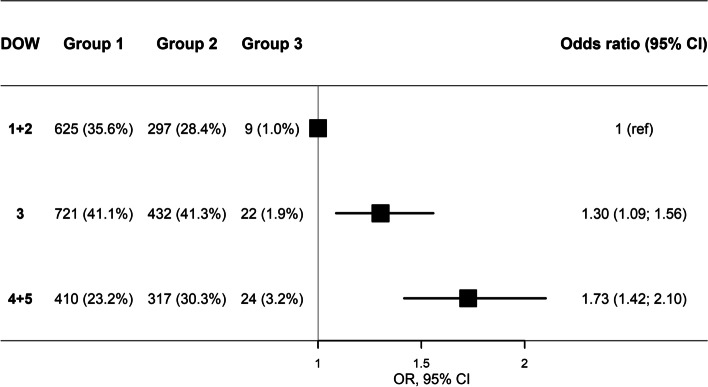
Odds ratio for association between DOW and more severe patient outcome (OR*, 95% CI, Forest plot)

The analysis adjusted for age and gender for association between DOW and receiving FTF as triage response had OR 1.57 (1.32–1.88) and 2.20 (1.81–2.68) for medium and high DOW, respectively, and these ORs were not different to the unadjusted analysis. The unadjusted analysis for association between increasing DOW and risk of hospitalization ≥24 h, with low DOW as a reference, showed an OR of 1.95 (95 CI 0.90–4.27) for medium DOW and OR 3.38 (95% CI 1.56–7.32) for high DOW. The effect changed to OR 1.98 (95% CI 0.91–4.33) and OR 3.33 (95% CI 1.53–7.21), respectively, after adjusting for age and gender. Ordinal logistic regression for association between increasing DOW and more severe patient outcome within 48 h of the call, showed an adjusted OR of 1.30 (95% CI 1.09–1.56) and 1.73 (95% CI 1.42–2.10) for medium and high DOW, respectively, Fig. 3.

As the qualitative analysis focuses on group 3, we performed a subgroup analysis on these patients, Table 2. The results were divided into three groups of DOW, to be more focused on the research aim of expressions of worry. The relative occurrence of older children was highest in the group of high DOW, and so was the number of patients referred to FTF directly after this first call.

**Table 2:** Patient characteristics of patients hospitalized ≥24 h (group 3), stratified by DOW. Values are expressed as numbers (n) and percentages (%)

	Low DOW *n* = 9 (16%)	Medium DOW *n* = 22 (40%)	High DOW *n* = 24 (44%)
**Gender**			
Boys	4 (44%)	11 (50%)	13 (54%)
**Age**			
Median (range)	2 (1–5)	1 (0–2)	2.5 (0–8.5)
0–2 years	5 (56%)	17 (77%)	12 (50%)
3–5 years	2 (22%)	2 (9%)	1 (4%)
6–11 years	2 (22%)	3 (14%)	11 (46%)
**MH1813 triage response**			
Telephone consultation	5 (56%)	5 (23%)	3 (13%)
Face-to-face consultation	4 (44%)	17 (77%)	21 (88%)

*DOW* Degree-of-worry, *MH1813* Medical Helpline 1813

### Qualitative strand

Out of the 55 children in group 3, we were able to locate the voice log for 54 calls. To answer our research aim, we focused the following analysis on manifestations of worry only. We identified two themes relating to expression of worry: *reason for worry* and *regaining control*, Table 3. The themes are elaborated below.

**Table 3:** Findings of content analysis related to expressions of worry in calls regarding severely ill children (Group 3: hospitalized ≥24 h)

Quotes (meaning units)	Themes, categories and codes
	**Theme: Reason for worry**
*“A couple of hours ago, at around seven o’clock, [name] suddenly got this really intense pain in her stomach (…) it happened really fast. Like out of the blue.”* (1695)	Category: Loss of control Codes: - Deterioration or symptom changing - New symptom occurred - Rapid onset - Treatment is not working as expected
*“Because I never really experienced that, I have three children, right, and I have never experienced any of them having this intense stomach pain.”* (2594)	Category: Previous experiences Codes: - Worried due to previous experiences - Illness unlike previous experiences - Chronic disease
*“-He was diagnosed with right-sided pneumonia, which he got prescribed antibiotics for.* *-Yes.* *-Eh, I don’t know, I don’t know how long I should like wait and see, but I think he has gotten worse, especially his breathing is worrying me.”* (2151)	Category: Illness manifestation Codes: - Breathing difficulty - High fever - Intense stomach pain - Infant
*“And coughing, it is hopefully just normal eh, cold or the flu, I don’t know* [short pause] *what to do”* (1585)	Category: Uncertainty Codes: - I don’t know what to do, I don’t understand
*“-I’ve been to the doctors twice with her today. This morning she was diagnosed with tonsillitis and* [interrupted] *-This morning, you said?* *-Yes, this morning. And then she got worse and worse* *(−Yes.)* *- and got troubled breathing so I went to the doctor again at 2 o’clock where she said that she could hear a bad pneumonia on the right side.* *(−Okay.)* *-So, we were asked to take some more antibiotics and she’d immediately get better. And she’s not, but* [interrupted] *-Well, ‘immediately’, that’s such a daft thing to say, I’m sorry but* [interrupted] *-It’s not because it should, she only said that she shouldn’t get worse. (2147)*	Category: Recent healthcare contact Codes: - Has been to the hospital - Contact to GP/MH1813
*“-But his respiratory rate is 42 and* [interrupted] *-That’s because he has a fever.* *-No, it’s not. Well yes, it’s natural that his respiratory rate is increased, but not that much. And he’s grunting and having retractions.* *-May I hear his breathing?* *-Yes, of course (…) I don’t know how much you could hear, but what worries me is his* [interrupted] *-May I hear it again? I will make a referral; I just want to hear it myself.”* (2166, caller [father] is a physician) *“-Yes, what seems to be the matter with your son?* *-He has* [interrupted] *-His name is [name]?* *-Yes, that’s right. He has a fever of 39.7 and* [interrupted] *-For how long has he had a fever?* *-He has had it since afternoon, noon today he has had fever, so I give him* (paracetamol) *a lot, but it doesn’t help, and he wheezes because* [interrupted] *-When did you last give him some* [paracetamol]*?* *-Eh, I gave him last time four hours ago.* *-Yes, eh.* *-Problem is also, he has asthma and he coughs a lot. I was at the GP today with him, but they say maybe he* [interrupted] *(…)* *-And I have given him the reliev-* [interrupted] *-And you have given him medicine?* *Yes, I give him acute* (broncodilator) [interrupted] (1588, caller [father] speaks with foreign accent, but quite understandable)	Category: Caller characteristics Codes: - Caller is the mother - Caller is the father - Caller is a healthcare professional - Caller belongs to minority group (potential language barrier)
	**Theme: Regaining control**
*“-Okay. And you don’t think it can wait until tomorrow, until our GP opens?* *-No, not, the reason why I think it can’t wait is that he’s so young and he has got these, what we call retractions, he uses all of his, his accessory muscles to breathe.* *-Okay.* *-Mm, so that’s why, and that, that shallow breathing that you’re describing, right, even if* *(−Yes.)* *-as you say, his general well-being seems good enough, but* [interrupted] *-Yes, because as I said, he’s happy, he’s lying and looking around, right, it’s just …* “(1590)	Category: Response to triage decision Codes: - Reluctance to going to the hospital - Caller still not in control - Caller has regained control

*GP* General practice, *MH1813* Medical Helpline 1813 (…): a section of the conversation has been omitted

#### Reason for worry

Contact to MH1813 was often caused by worry. Callers worried if there were changes in the child’s condition or by the addition of new symptoms, particularly if changes happened quickly. If initiated treatment, typically antibiotics or bronchodilators, did not have the expected effect, callers worried. These worries could lead to a feeling of loss of control.

Previous experiences affected parents’ worry as well. Either because the symptom was serious and had led to urgent care earlier, or because the caller was unfamiliar with the symptom. Some parents reported that their child had a chronic disease such as asthma. Parents who were familiar with the current condition used medical terms to describe the child’s state.

The callers were worried if their child had trouble breathing. High fever was another reason for worry and reason for contact. There were many calls concerning abdominal pain. These calls were longer than the other calls because there was more exchange of information. Parents were overwhelmed by the intensity of pain in the child, causing worry. Furthermore, special attention was given in calls where the child was very young.

Several callers mentioned having had contact to GP, or rarely, to MH1813, during the same episode of illness. Sometimes the parent told the call-handler that they had been instructed to call if they experienced worsening or lack of improvement. Furthermore, if the child had been to a hospital recently, the call-handler would quickly refer to renewed FTF assessment. It was important to the call-handler if the child had already been assessed by a healthcare professional as this could be a sign that the child was more severely ill.

Lastly, the characteristics of the caller was of importance to the communication and to the call-handlers’ perception of caller proficiency. The call was made by the mother in 34 cases, by the father in 18 cases and by a friend or relative in two cases. These two latter cases were due to language barriers, where the parents allegedly could not communicate sufficiently well in Danish.

In several of the calls placed by fathers, the mothers were in the background and contributed substantially to the conversation. This was not true for the opposite situation. It was of importance if the caller was a healthcare professional, as was the case in four calls, as these calls were noticeably shorter and the parents efficiently reported the vital symptoms and treatment given. Even though the parent was a healthcare professional, some call-handlers found it important to ask relevant questions and to present their own assessment of the situation. In a few calls, the caller would display some annoyance when their professionalism was not duly respected, and vice versa. But in general, these calls were characterized by efficient communication and interaction.

Several calls (*n* = 5) were made by parents talking with an accent. Experiencing a language barrier had profound impact on the conversation and its content, as these calls often distinguished themselves from the other calls. Firstly, these parents did not receive as many questions regarding the child’s general state and accompanying symptoms, such as fluid intake, neck stiffness and rash. Secondly, these parents were interrupted or ignored more often, especially in the beginning of the call, where information-giving and -gathering take place. There were also some misunderstandings due to misinterpretation of words.

#### Regaining control

The parents had different responses to the triage decision. A few parents were surprised to hear that the call-handler recommended them to go to the hospital or asked if it really was necessary that they went to a hospital right now. Even a few very worried parents were reluctant to go to the hospital right away. Conversely, several parents who were triaged to staying at home were not sufficiently comforted by the call-handler and did not regain control after the call. They tried to show their concern by pausing, sighing or using a despairing tone of voice. Some parents repeated questions concerning e.g. treatment continuation when the call-handler tried to end the call. Callers regaining control accepted the triage outcome and displayed no hesitation or dissatisfaction and did sometimes explicitly state that they were happy with the outcome.

### Mixed methods analysis

Through the mixed methods analysis we wished to expand knowledge of DOW by merging quantitative and quantitative data. Firstly, we undertook transformation of data, where the occurrence of codes found during qualitative analysis were transformed into numbers and are presented as percentages, distributed on the three different DOW-groups (Table 4).

**Table 4:** Distribution of codes in calls regarding seriously ill children (Group 3: hospitalized ≥24 h), stratified by varying DOW, *n* = 54. Values are given as percentages (%)

Themes, categories and codes related to content regarding worry	Low DOW	Medium DOW	High DOW
**Reason for worry**			
Loss of control			
Deterioration or symptom changing	5.3%	42.1%	52.6%
New symptom occurred	36.4%	27.3%	36.4%
Rapid onset	0%	40%	60%
Treatment not working as expected	20%	30%	50%
Previous experiences			
Worried due to previous experiences	0%	0%	100%
Illness unlike previous experiences	28.6%	42.9%	28.6%
Chronic disease	55.6%	22.2%	22.2%
Illness manifestation			
Breathing difficulty	0%	28.6%	71.4%
High fever	33.3%	33.3%	33.3%
Intense stomach pain	7.7%	30.8%	61.5%
Young child	0%	40%	60%
Uncertainty			
I do not know what to do, I do not understand	26.3%	42.1%	31.6%
Recent healthcare contact			
Has been to the hospital	20%	20%	60%
Contact to GP/MH1813	12.5%	18.8%	68.8%
Caller characteristics			
Caller is the mother	20.6%	41.2%	38.2%
Caller is the father	12.5%	37.5%	50%
Caller is a healthcare professional	0%	83.3%	16.7%
Caller belongs to minority group (potential language barrier)	20%	0%	80%
**Regaining control**			
Response to triage decision			
Reluctance to going to the hospital	25%	50%	25%
Caller still not in control	50%	0%	50%
Caller has regained control	15.7%	41.2%	43.1%

*GP* General practice, *MH1813* Medical Helpline 1813

When concentrating on the reason for worry, parents who were worried to a high or medium degree focused on deterioration and ineffective treatment. Children receiving antibiotics were uniquely found in the high DOW-group. These parents more often considered previous experiences, such as prior hospitalization due to similar symptoms, or, conversely, lack of experience with similar symptoms and they often called due to trouble breathing or stomach pain. Calling about a very young child was associated with a higher DOW. On the other hand, parents with children with chronic diseases had low DOW and did display less loss of control possibly because they had less uncertainty concerning the current situation.

Among those with highest DOW, several parents mentioned having had previous contact with their GP, or rarely, MH1813. Mothers were the most prevalent callers and they rated DOW lower than fathers who were more often in the medium or high DOW-group. All calls made by a healthcare professional parent except one were scored as medium DOW. Several calls were made by parents with an accent, and these calls were mostly (80%) found in the high DOW-group.

Parents in the different DOW-groups reacted somewhat differently to the triage outcome. Parents being surprised to get referred to the hospital were most prevalent in the low DOW-group. Most parents were reassured by the call, but parents who did not regain control were found in the low or high DOW-group.

**Fig. 4:**
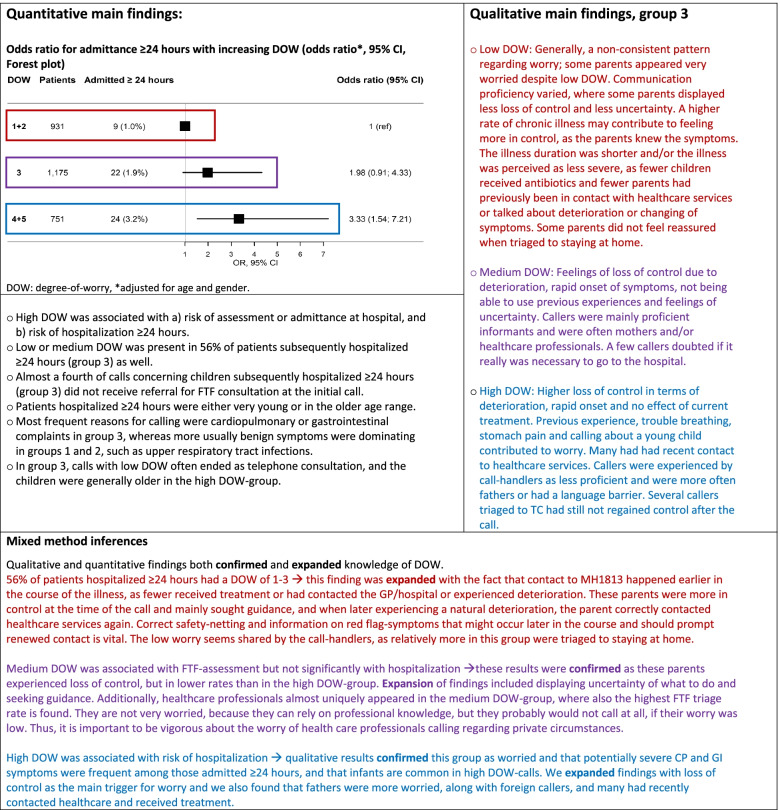
Joint display of mixed methods analysis and results

We constructed a side-by-side joint display which presented the most important findings, and this common visual presentation enabled the creation of meta inferences, Fig. 4. Quantitative analysis showed that high DOW was associated with a) risk of assessment or admittance at hospital, and b) risk of hospitalization ≥24 h. Qualitative findings both confirmed and expanded knowledge of DOW, Fig. 3. Contact to MH1813 occurred earlier in the illness trajectory in calls with low DOW, whereas high DOW was associated with loss of control and more severe symptoms. Parents with medium DOW experienced less loss of control and often sought guidance.

## Discussion

### Summary of results

In this mixed methods study we investigated parents’ expression of worry in calls regarding acutely ill children to a medical helpline. The quantitative and qualitative results confirmed and expanded each other. High parental DOW was significantly associated with hospitalization ≥24 h. Loss of control was the major trigger for worry in this group, and was provoked by deterioration or changing symptoms, rapid onset or treatment not working. Among the parents with children getting hospitalized ≥24 h but rating their DOW as low, we found that the contact occurred earlier during the illness. It is possible that this group of parents did have a notion of something being different than usual even that early during the illness, as they chose to contact MH1813 despite, at least subjectively, mild symptoms. Additionally, in the medium DOW-group, which was associated to face-to-face consultation but not hospitalization ≥24 h, there was a high proportion of health care professionals, and their decision to call MH1813 seemed well-considered and not taken lightly. Lastly, we found that parents with foreign background and speaking with an accent often rated their DOW as high, but these calls were characterized by the caller often getting interrupted or overheard, and they received less questions regarding the general state and accompanying symptoms than other callers. Some of these calls were ended as telephone consultations, and the parent did not seem reassured by this outcome.

### Comparisons with existing literature

Our main finding that high DOW is associated with the child being hospitalized ≥24 h is in accordance with several studies where worry has been found as a principal reason for parents’ decision to contact health care services [10, 11, 34]. This worry has been expressed on a range from “need for reassurance” to “perceived condition to be life threatening”. This sense of worry and thus need for contact, might be interpreted as a form for gut feeling, and it has not been well studied, neither has the clinician’s sense of worry or notion that “something is wrong” in the field of pediatrics. Studies have shown that parents tend to worry less the older the child gets and if they have more than one child [16, 17, 35, 36]. It might seem like parents gradually become more experts in judging when there is reason to worry. That is, experience could affect the usefulness of the gut feeling, or sense of worry. In the study previously referred to, where the clinician’s feeling of “something is wrong” and the similar parental notion of “illness is different” was of great importance to identifying severely ill children, the authors were clear about the fact that they did not know what this gut feeling was based upon [20]. Interestingly, clinicians’ professional experience more or less than 10 years did not alter this finding. Qualitative findings indicate that gut feeling among GPs, defined as “something is wrong here” in the context of identifying child abuse, is experienced as a valuable diagnostic tool [37]. As such, subjective feelings of unease, expressed as worry or a bad gut feeling, seem to be of importance when assessing children in different health care circumstances, but more studies are needed.

We found that perceived loss of control was the trigger to calling MH1813. As parents’ degree-of-worry and sense of urgency not always align with the views of health care professionals, potential conflicts may be at risk. Another study investigated parental calls to a medical helpline, and how call-handlers responded to parents’ concern [38]. They found a general lack of open questions, exploration of patients’ motives, expectations and worries, as well as a paucity of searching for if concordance and reassurance had been found at the end of the call. This in spite of, as the authors concluded, that patient-centeredness and open questioning are part of guidelines in many call-centers, and parents’ desires to be heard, respected and getting their concerns relieved are well-known [38]. Our study confirms the paucity of patient-centeredness. A study regarding calls with subsequent malpractice claims showed that open questions provide more medical information without increased time use [39]. Thus, a higher degree of open questioning during the call as well as an assessment of if the caller has regained control of the situation at the end of the call would be desirable to ensure sufficient parental reassurance. Several studies have focused on medical appropriateness of contacts to OOH services in both adults and children, and it is generally concluded that we should move away from the traditional medical focus and instead aim at including and assisting callers in the decision-making process, as the disparity between medical professionals’ and callers’ opinion on urgency is sometimes profound [5, 22, 35, 40]. In calls assessed as medically inappropriate by call-handling GPs, there was a significant association to unfulfilled patient expectations [40]. Furthermore, previous encounters with healthcare professionals have profound impact on parents’ help-seeking [41]. Felt or enacted criticism teaches parents informal social rules of how they are expected to behave, and such encounters may leave parents feeling unable to handle the present situation and still feeling worried. On the other hand, positive encounters may induce increased parental empowerment and a validation of their decision to contact health care services [41]. Finally, it is natural to worry when your child is ill, and parents themselves have stated that it is difficult to act rationally when they are feeling emotional concern [16, 17].

Furthermore, some of the factors we found associated with high DOW have been explored in other studies. We found that fathers made fewer calls but were more worried. In Sweden, with a healthcare system resembling the Danish model, mothers most often contacted *Swedish Healthcare Direct* when a child was ill, but fathers received FTF response more often than mothers [42]. The authors concluded that more studies regarding gendered assumptions are needed and that recruitment of male call-handlers might be beneficial.

Calls from foreign callers have been experienced as challenging by call-handlers previously [3]*.* Apart from communication problems due to language issues, these callers have been described as exaggerating and demanding, and the calls tend to feel time-consuming and requiring much energy. Non-Western immigrants have been found to have an increased use of OOH services as well as a higher degree of perceived urgency, higher expectations of physical examination and receiving a prescription [43, 44]. Possible explanations presented were impaired accessibility to GP, low health literacy and different expectations to doctors’ consultations due to experiences in their country of origin. Among the adult study population from the original DOW-study, non-Western immigrants were found to more often rate their DOW as high, and this could possibly be explained by lower health literacy and socio-economic status in this group [45].

Not much is known about parents with low DOW who choose to contact OOH services anyway. However, it has been shown several times that convenience and practical reasons play major roles in callers’ decisions to contact OOH services [16, 35, 46]. It is experienced as beneficial that you can get an appointment easier and faster in OOH services or PEDs than in GPs, which might be needed to be able to go to work the next day, as the GP offices are closed when parents get off work. The availability of more specialized staff, tests and treatments at hospitals is also desired by some parents [35]. Increased focus on primary care as a measure to take pressure off hospitals was suggested in a systematic review studying unscheduled pediatric healthcare visits [35]. It was also suggested as a preventive measure to counteract high use of urgent health care by young children in the Copenhagen Region, along with a nurse hotline for questions about sick children [19].

### Implications for clinical use and future research

Introducing DOW as part of the triage process may ensure inclusion of the patient’s perspectives, and especially high DOW can be used to find the potentially most ill patients, a task that might be challenging considering the high number of mildly ill children at call-centers. DOW can be used to improve communication by exploring the caller’s fears and expectations. If the call-handler and the caller hence are able to reach a suitable triage outcome that reassures the caller and matches the medical need, both would be more satisfied with the call.

Over one third of callers rated their DOW as low. These parents could possibly have been reassured by contacting their GP, but as the availability of the GP is perceived as limited by the parents, a strengthened primary care with opening-hours adjusted to the needs of the patients would be a possible solution. Even so, we found that many parents with children later hospitalized ≥24 h rated DOW as low-to-medium at the initial call and these children often displayed mild symptoms. Therefore, it is important to instruct parents in what to observe and when to contact the healthcare services again. Some might need a face-to-face consultation at a later time due to natural deterioration. The advice included in the safety-netting must be customized to suit the individual call, according to factors such as child’s age, parents’ previous experiences, and the nature of the present symptom(s), but common red-flag symptoms should be always be included, e.g. impaired contact, lack of fluid intake and abnormal respiratory rate.

We found a general lack of open questions to the parents and extra focus should be spent on this factor. As loss of control was the main trigger of worry, and thus elicited contact, it is crucial to reassure the caller sufficiently to prevent re-presentation and to empower the parents in feeling able to care for their mildly ill children at home.

Lastly, we found that immigrant parents were more worried than other parents. To better understand this group of parents, further studies of their expectations and health literacy are needed. Until then, improved quality of communication and call-handling of this group should be a focus. These parents should inarguably receive the same prudent call-handling as others to ensure that no important symptoms are uncovered.

### Strengths and limitations

The strengths of the study include the high-quality Danish registers ensuring complete and reliable outcome registration. Furthermore, due to the set-up with study invitation and -participation immediately before the conversation with the call-handler, there was no risk of recall bias, and a large number of patients of a broad variety could be included. The trustworthiness of the study was increased by investigator triangulation, and in the final steps of the qualitative analysis multiple researchers discussed findings to agree on the best interpretation of data. Credibility was increased by using a well-established qualitative research method.

There are some limitations to this study. Firstly, only 33% of the total number of callers in the study period chose to participate. A non-response bias analysis revealed no differences in age, gender or triage outcome between responders and non-responders in the DOW population, but selection bias cannot be completely ruled out [23]. Secondly, for the qualitative analysis we used recorded conversations that were not conducted with research in mind. This might have a negative impact on the relevance of the content and the audio quality, but the calls inarguably reflect clinical reality. As always, the generalizability of qualitative studies is debatable, but we have provided rich information on setting and design to enable other researchers to assess this dimension before transferring results to other contexts. Lastly, we chose to use length of hospitalization as a proxy for illness severity. Patients with serious symptoms that were successfully treated within 24 h were thus not included in group 3; the most ill patients. The association between DOW and hospitalization could have been more significant if these patients too had been included.

## Conclusion

Degree-of-worry ratings can be used at medical call-centers to include parents’ perspectives, and high DOW can be used to find the most severely ill children, an otherwise challenging task. Our findings show that extra focus should be directed at using open-ended questions and at ensuring parents’ control of the present situation, and lastly, at improving communication and call-handling in calls from immigrant parents.

## Data Availability

The datasets generated and/or analyzed during the current study are not publicly available due to data containing personal information and information on health status but are available from the corresponding author on reasonable request.

## Abbreviations

CI: Confidence interval
DOW: Degree-of-worry
EMS: Emergency Medical Services
FTF: Face-to-face
GP: General practice
IQR: Interquartile range
MH1813: Medical Helpline 1813
OOH: Out-of-hours
OR: Odds ratio
PED: Pediatric emergency department
TC: Telephone consultation
